# Monitoring SARS-CoV-2 Using Infoveillance, National Reporting Data, and Wastewater in Wales, United Kingdom: Mixed Methods Study

**DOI:** 10.2196/43891

**Published:** 2023-11-23

**Authors:** Jordan P Cuff, Shrinivas Nivrutti Dighe, Sophie E Watson, Rafael A Badell-Grau, Andrew J Weightman, Davey L Jones, Peter Kille

**Affiliations:** 1 School of Biosciences Cardiff University Cardiff United Kingdom; 2 School of Natural and Environmental Sciences Newcastle University Newcastle-upon-Tyne United Kingdom; 3 Division of Genetics, Department of Paediatrics University of California, San Diego La Jolla, CA United States; 4 School of Natural Sciences Bangor University Bangor United Kingdom

**Keywords:** COVID-19, Google Trends, infodemiology, quantitative reverse transcription polymerase chain reaction, RT-qPCR, wastewater, infoveillance, public health care, health care statistics, correlation analysis, analysis, public health, online health, eHealth, public interest

## Abstract

**Background:**

The COVID-19 pandemic necessitated rapid real-time surveillance of epidemiological data to advise governments and the public, but the accuracy of these data depends on myriad auxiliary assumptions, not least accurate reporting of cases by the public. Wastewater monitoring has emerged internationally as an accurate and objective means for assessing disease prevalence with reduced latency and less dependence on public vigilance, reliability, and engagement. How public interest aligns with COVID-19 personal testing data and wastewater monitoring is, however, very poorly characterized.

**Objective:**

This study aims to assess the associations between internet search volume data relevant to COVID-19, public health care statistics, and national-scale wastewater monitoring of SARS-CoV-2 across South Wales, United Kingdom, over time to investigate how interest in the pandemic may reflect the prevalence of SARS-CoV-2, as detected by national testing and wastewater monitoring, and how these data could be used to predict case numbers.

**Methods:**

Relative search volume data from Google Trends for search terms linked to the COVID-19 pandemic were extracted and compared against government-reported COVID-19 statistics and quantitative reverse transcription polymerase chain reaction (RT-qPCR) SARS-CoV-2 data generated from wastewater in South Wales, United Kingdom, using multivariate linear models, correlation analysis, and predictions from linear models.

**Results:**

Wastewater monitoring, most infoveillance terms, and nationally reported cases significantly correlated, but these relationships changed over time. Wastewater surveillance data and some infoveillance search terms generated predictions of case numbers that correlated with reported case numbers, but the accuracy of these predictions was inconsistent and many of the relationships changed over time.

**Conclusions:**

Wastewater monitoring presents a valuable means for assessing population-level prevalence of SARS-CoV-2 and could be integrated with other data types such as infoveillance for increasingly accurate inference of virus prevalence. The importance of such monitoring is increasingly clear as a means of objectively assessing the prevalence of SARS-CoV-2 to circumvent the dynamic interest and participation of the public. Increased accessibility of wastewater monitoring data to the public, as is the case for other national data, may enhance public engagement with these forms of monitoring.

## Introduction

The COVID-19 pandemic has given rise to a range of public responses that have dynamically driven the cooperation of the public with governmental guidance and public recognition of the need for regular testing. Health care systems have been stretched beyond capacity by sudden, large-volume influxes of patients following sometimes unpredictable waves of the virus [1]. There is a pressing need for local, national, and global adaptability to manage these outbreaks of the disease to minimize the impact on health care systems, the first requirement of which is the stringent collection of reliable and accurate data on viral prevalence [2].

Many strategies have been used to monitor SARS-CoV-2, for example, self-reporting [3] and participatory surveillance [4-6], including through the use of platforms such as accessible phone apps [7]. Surveys and self-reporting, achieved through participatory surveillance and even active crowdsourcing strategies, have proven highly effective in monitoring symptoms such as loss of taste [8]; participatory surveillance platforms such as this have been a crucial component of monitoring in partnership with the public [8,9]. Relying on surveys and personal testing data, however, allows only a reactive approach to mitigating the health care burden imposed by COVID-19, which is often too little, too late to mitigate the heavy case numbers and death tolls. Case data, while sometimes collected by standardized surveys, can otherwise depend on self-reporting by the public, many members of which may not self-test given poor access to tests, may not feel obliged due to asymptomatic cases, or may receive false negative results. Others may unreliably or even dishonestly report the results of tests given the restrictions that a positive test for COVID-19 imposed [10], or they may be disenfranchised with the efforts to reduce the prevalence of the disease given the overwhelming extent of misinformation in circulation [11].

Search engine use has been explored as a means for ascertaining the prevalence of diseases [12,13], but this method is not infallible and its accuracy over time must be assessed in different epidemiological contexts [14,15]. Such data could anecdotally track COVID-19 or specific related symptoms [16-19] but the public searching for particular character strings cannot be directly ascribed to the prevalence of the disease. This “infoveillance” does, however, facilitate analysis of public interest in subjects such as the pandemic [11,20], which can be an important factor in health care management and the pandemic response. Infoveillance can be integrated into interdisciplinary frameworks such as “One Health” [21,22] and, more specifically, “One Digital Health” [23], which aim to view health care matters more holistically, particularly the interaction between human and veterinary health and its implications for zoonotic diseases, but also the environmental dimension of disease occurrence and transmission.

Given the latency of surveys and testing by the public, and the potential inaccuracies of infoveillance approaches, objective means for disease surveillance without the requirement of public participation have become increasingly important throughout the COVID-19 pandemic. The presence of coronaviruses and other human pathogenic viruses in human feces and their subsequent presence in urban wastewater is a long-established tool for assessing disease prevalence within a community [24,25]. This approach provides a noninvasive means for assessing SARS-CoV-2 prevalence across whole populations via wastewater [25-31]. The monitoring of wastewater has provided a robust and accurate means of assessing the population-level prevalence of COVID-19, facilitating some prediction of health care burden before symptoms arise [32]. Wastewater monitoring circumvents several barriers preclusive to accurate testing data such as hesitancy, the availability of testing, asymptomatic patients, and socioeconomic or cultural barriers by passively sampling from whole communities [10,33]. The efficacy of this approach does not depend on public participation, possibly leading to some inconsistencies with national testing statistics. A strong positive correlation between direct testing, wastewater monitoring data, and public interest in the pandemic has been demonstrated [34], but the dynamic relationship between these data and how public interest dictates the accuracy of monitoring data are still poorly characterized.

Here, we compare public interest in the pandemic through search engine use data against wastewater SARS-CoV-2 surveillance data and nationally reported statistics over time to assess how public interest dictated the relationship between disease prevalence and reporting over a year of the COVID-19 pandemic in South Wales, United Kingdom. This study also explores the efficacy of wastewater monitoring and infoveillance as means for assessing the national state of the pandemic, how these relationships change over time, and how they could inform predictions of case numbers for streamlined monitoring.

## Methods

### Wastewater Monitoring

Since mid-September 2020, wastewater samples were collected every Monday, Wednesday, and Friday from Cardiff Bay, Newport Nash, Llanfoist, Ponthir, Ogmore, Cog Moors, Swansea Bay, and Gowerton wastewater treatment plants, and samples from Carmarthen and Haverfordwest were collected every Wednesday. Samples were transported on ice in a cooler box to designated wastewater processing facilities at Cardiff University. The processing of samples was based on Farkas et al [35]. From each site, 200 mL of wastewater was spun at 3000×g for 30 minutes, and 150 mL of supernatant was neutralized to pH 7-7.4 using 1 M NaOH. The supernatant was incubated with 50 mL of 40% PEG and 8% NaCl overnight. Samples were then spun at 10,000×g for 30 minutes and the pellet was dissolved in 500 µL of PBS (pH 7.4). Of the dissolved pellet, 100 µL was spiked with 10,000 copies of synthetic murine norovirus DNA to check the extraction efficiency. Subsequent nucleic acid extraction and amplification took place in the COVID-19 testing facilities at Cardiff University. Total RNA was extracted using the methodology published by Oberacker et al [36]. Total RNA was eluted in 100 µL of nuclease-free water. For SARS-CoV-2 detection, 4 primer sets published by the US Centers for Disease Control and Prevention (CDC), Charité, and Hong Kong University [37] were used for quantitative reverse transcription polymerase chain reaction (RT-qPCR). Primer sets N1 and N2 target different regions of the nucleocapsid (N genes); E_Sarbeco and ORF1b target the SARS-CoV-2 E and nsp14 genes, respectively. For the controls, a set of primers that target virus crAssphage [38] (which is present in human fecal material) and murine norovirus [39] (which was used to assess extraction efficiency) were selected (Table 1). Samples were run in triplicate on Fast 384-well plates (Applied Biosystems) using QuantStudio 7 Flex (Applied Biosystems). A 10 µL RT-qPCR reaction was performed containing 4 µL of extracted RNA template, 5 µL of Luna Universal Probe One-step Reaction Mix (2X; NEB), 0.04 µL of each primer set (100 µM), 0.02 µL of fluorescent probe (100 µM), 0.5 µL NEB Luna reverse transcriptase (20X), and 0.4 µL nuclease-free water. The reverse transcription (RT) was carried out at 55 °C for 10 minutes, followed by polymerase activation at 95.0 °C for 1 minute and 40 cycles of denaturation, annealing, and extension at 95.0 °C for 10 seconds and then 60.0 °C for 1 minute, respectively. Serial dilutions of the heat-inactivated SARS-CoV-2 viral standards were run on every PCR plate to generate standard curves used to quantify the copies of SARS-CoV-2 genes. Additionally, RT-qPCR runs were validated by positive (Qnostics, SCV2QC01-QC) and negative controls (nuclease-free water). Resultant data were normalized to account for population size in each area, and to correct for dilution as described by Wilde et al [40].

**Table 1:** The quantitative polymerase chain reaction (qPCR) primers used for wastewater monitoring.

Assay	Target gene	Sequences (5’-3’)
E_Sarbeco	E	F- 5’-ACAGGTACGTTAATAGTTAATAGCGT-3’ R- 5’-ATATTGCAGCAGTACGCACACA-3’ P- 5’-HEX-ACACTAGCCATCCTTACTGCGCTTCG- IBFQ-3
ORF1b	nsp14	F-5’-TGGGGYTTTACRGGTAACCT-3’ R-5’-AACRCGCTTAACAAAGCACTC-3’ P- 5’-FAM-TAGTTGTGATGCWATCATGACTAG- IBFQ-3’
N1	Nucleocapsid	F- 5’-GACCCCAAAATCAGCGAAAT-3’ R- 5’-TCTGGTTACTGCCAGTTGAATCTG-3’ P-5-HEX- ACCCCGCATTACGTTTGGTGGACC- IBFQ-3’
N2	Nucleocapsid	F- 5’-TTACAAACATTGGCCGCAAA-3’ R- 5’-GCGCGACATTCCGAAGAA-3’ P- 5’-FAM- ACAATTTGCCCCCAGCGCTTCAG-IBFQ-3’
crAssphage	Q56	F- 5’-CAGAAGTACAAACTCCTAAAAAACGTAGAG-3’ R- 5’-GATGACCAATAAACAAGCCATTAGC-3’ P- 5’-HEX- AATAACGATTTACGTGATGTAAC-IBFQ-3’
MNV^a^	—^b^	F- 5’-CCGCAGGAACGCTCAGCAG-3’ R- 5’-GGYTGAATGGGGACGGCCTG-3’ P-5’-FAM- ATGAGTGATGGCGCA- IBFQ-3’

^a^MNV: murine norovirus.

^b^Not applicable.

### National Statistics and Search Volume Data Extraction

This study concerns 2 periods: the primary study period (between the weeks of October 11, 2020 and October 31, 2021; the focus of all analyses and visualizations aside from comparison with model-based predictions described below) and the full study period (the primary study period with extension up to July 17, 2022 to facilitate comparison of real-world data with model-based predictions). All data were generated or extracted to encompass the full study period. National statistics on the daily number of COVID-19 cases, deaths, and vaccinations in Wales were extracted from the UK government’s COVID-19 data portal for the full study period [41]. Case data were new cases by publish date (ie, the number of new cases reported since the previous update; API=“newCasesByPublishDate”). Death data were new daily national statistics office deaths by death date (ie, daily numbers of deaths of people whose death certificate mentioned COVID-19 as one of the causes; API=“newDailyNsoDeathsByDeathDate”). Vaccine data were new vaccines given by publish date (ie, daily numbers of new vaccines [all doses] given; API=“newVaccinesGivenByPublishDate”). These data can be downloaded via a permanent download link [41].

Search volume data were extracted from Google Trends. These data provide a proxy for public interest in or response to the extent of the COVID-19 pandemic. The data extracted from Google Trends are relative search volumes (RSVs) for predetermined search terms, allowing comparison of search rates for different terms via Google, the most widely used internet search engine. These RSVs are presented for each date of a given period within a given country, nation, or region and are normalized relative to the highest search volume peak in that search batch in the time period specified (this peak is represented as a search volume of 100%). Search volumes were releveled so that the highest peak in the primary study period was represented by “100” and any higher peaks across the full study period exceeded 100 to reflect the limitations of making real-time predictions from existing data. Given the representation of numbers less than 1 as “<1” by Google Trends, all RSVs of “<1” were converted to 0 to facilitate quantitative comparison.

Search terms were selected based on their broad relevance throughout the study period and the high volume of searches generated during that period. These included “COVID lockdown,” “COVID rules,” “COVID symptoms,” “COVID test,” and “COVID vaccine.” “COVID” was included in each search term to ensure relevance to the COVID-19 pandemic; “COVID” was selected over “coronavirus,” “SARS-CoV-2,” and other variations due to the greater prevalence of searches related to this string, and its inclusion within other search strings such as “COVID-19”.

### Statistical Analysis

Statistical analyses and plotting of data were carried out using R (version v4.0.3; R Core Team) [42] and all data and code are openly available [43]. Since wastewater sites were sampled weekly, all data were averaged first by site and then by week. Wastewater quantitative polymerase chain reaction (qPCR) data were log-transformed to improve model fit and visualization. Data were processed and aggregated using *tidyverse* packages for reproducibility [44].

Correlations between search volumes; wastewater SARS-CoV-2 prevalence; and nationally reported cases, deaths, and vaccinations were tested using Spearman ρ rank correlation via the *rcor* function of the *Hmisc* package [45]. To facilitate the assessment of correlation, week dates were transformed into successive study weeks (ie, cumulative weeks of the study). The data were identified as nonnormally distributed via Shapiro-Wilk tests, so nonparametric correlation analyses were selected. The output was visualized in a correlogram via the *corrplot* function of the *corrplot* package [46], with colors to denote the strength of correlations assigned via the *viridis* package [47].

To assess how RSV for the selected search terms changed with differences in the number of COVID-19–related cases, deaths, and vaccines and the estimated prevalence of COVID-19 in wastewater, a multivariate linear model (MLM) was built via *manylm* in the *mvabund* package [4]. The dependent variable comprised the RSVs, log-transformed (log[n+1]) to achieve normality, and the independent variables were week; national cases, deaths, and vaccinations; and 2-way interactions between study week and each of the other variables. For visualization via line plots, data were releveled so that their minimum and maximum values were 0 and 100, respectively. These normalized search volume, wastewater, and government data were plotted against time using the *ggplot2* package [48], with colors assigned via the *paired* palette in the *RColorBrewer* package [49] and data lines smoothed using the *loess* method.

Pairwise plots were generated for reported case data, qPCR data, and RSVs from each of the Google Trends search terms separately using *ggpairs* from the *GGAlly* package. Linear models (LMs) were generated with the number of reported cases as the dependent variable and, in a separate model for each, the qPCR and Google Trends data as independent variables. The *predict* function was used to make interpolated predictions of case numbers across the primary study period and extrapolated predictions of case numbers beyond the primary study period for the remainder of the full study period. These predicted case numbers were plotted against the reported case numbers, and a correlation analysis was carried out as described above. A generalized linear model (GLM) with a Gaussian error family was built with reported cases as the dependent variables and predicted case numbers, time, and pairwise interactions between predictions and time as independent variables.

### Information Sources and Reliability

Wastewater monitoring data were generated by the authors of this study at Cardiff University as part of the Welsh government–funded WEWASH project. The national statistics on COVID-19 cases, deaths, and vaccinations were extracted from the UK government’s COVID-19 data portal [41], which is internationally recognized as a reputable source used for national reporting, scientific research, and public awareness. The Google Trends data should be reliable as indicators of Google use since they are collected by Google based on the input of users of their service.

## Results

**Figure 1:**
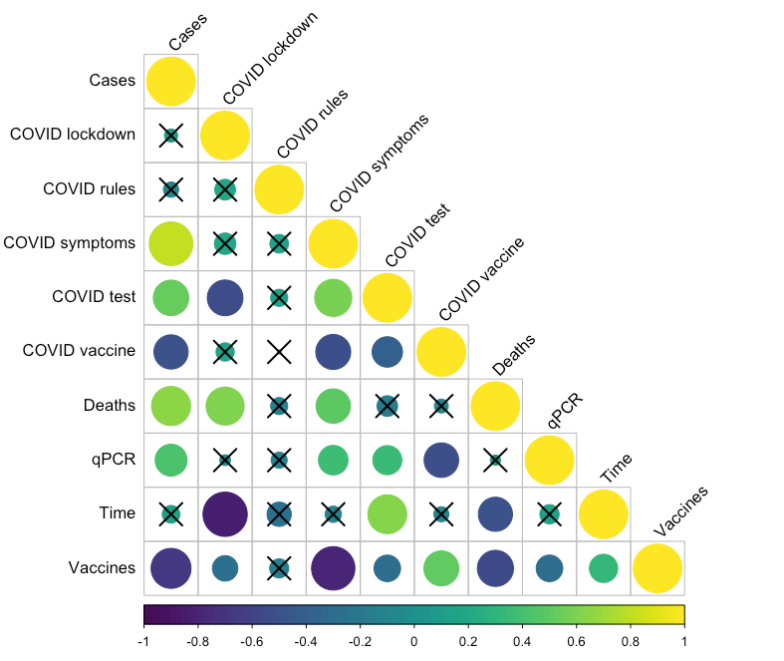
Correlogram of time (study week, ie, progressive number of weeks into the study period), Google Trends search volumes (variables starting with “COVID”), nationally reported cases, deaths and vaccinations, and qPCR-based wastewater SARS-CoV-2 RNA prevalence. Circle size and color (purple, through teal to yellow—denoting negative through neutral to positive) indicate the extent and directionality of the correlation. Crossed-out circles are those for which correlations were not significant. qPCR: quantitative polymerase chain reaction.

Overall, significant correlations were identified between many of the variables (Figure 1 and Table S1 in Multimedia Appendix 1). Notably, wastewater SARS-CoV-2 RNA prevalence significantly positively correlated with the number of reported cases (Spearman ρ=0.428; *P*=.001) but did not correlate with the number of reported deaths (Spearman ρ=0.044; *P*=.75). Of the search terms included, wastewater prevalence positively correlated with “COVID symptoms” (Spearman ρ=0.369; *P*=.005) and “COVID test” (Spearman ρ=0.356; *P*=.007) and significantly negatively correlated with “COVID vaccine” (Spearman ρ=–0.504; *P*<.001). The number of reported cases, however, positively correlated with both “COVID symptoms” (Spearman ρ=0.805; *P*<.001) and “COVID test” (Spearman ρ=0.531; *P*<.001) but negatively correlated with “COVID vaccine” (Spearman ρ=–0.495; *P*=.001). All search terms except “COVID rules” significantly negatively correlated with national vaccinations (all *P*<.05; Table S1 in Multimedia Appendix 1).

**Figure 2:**
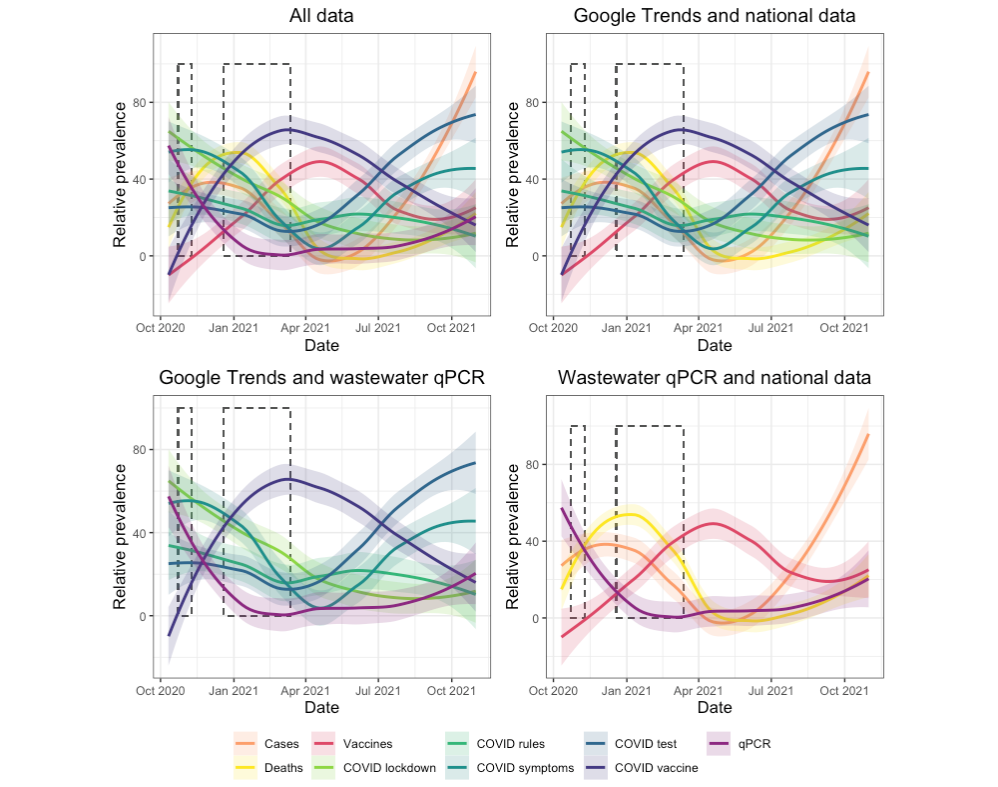
Relative search volumes extracted from Google Trends compared against nationally reported data and qPCR-based estimates of prevalence for SARS-CoV-2 in wastewater. All values are normalized so that the maximum value for each variable is 100. Lines are loess-smoothed curves, thus representing the overall trend, and do not always represent the most extreme (eg, maximum) values. Dashed rectangles represent periods of national lockdown in Wales for reference. Wastewater qPCR-based SARS-CoV-2 prevalence is given in light purple, Google Trends data are given in green or blue, and national data are given in orange or red or purple. A figure containing nonsmoothed trends is presented in Figure S1 in Multimedia Appendix 1. qPCR: quantitative polymerase chain reaction.

Search volumes were significantly related to several of the independent variables and their interactions (Table 2 and Figure 2), comprising wastewater SARS-CoV-2 prevalence (MLM: *F*_1,54_=34.89; *P*=.002); time (MLM: *F*_1,53_=120.89; *P*=.002); national COVID-19 cases reported (MLM: *F*_1,52_=117.77; *P*=.002); national COVID-19–related deaths reported (MLM: *F*_1,51_=65.84; *P*=.002); national COVID-19 vaccines administered (MLM: *F*_1,50_=54.31; *P*=.002); and the interactions between time and national COVID-19 cases (MLM: *F*_1,48_=46.32; *P*=.002), time and national COVID-19 deaths (MLM: *F*_1,48_=26.09; *P*=.004), and time and national vaccinations (MLM: *F*_1,46_=15.10; *P*=.02). The interaction between time and wastewater SARS-CoV-2 RNA prevalence (MLM: *F*_1,49_=0.77; *P*=.97) was not significantly related to RSVs.

**Table 2:** Univariate results from the multivariate linear model results for search volume data analyzed against time (progressive study weeks); wastewater SARS-CoV-2 RNA prevalence; nationally reported COVID-19 cases, deaths, and vaccines; and 2-way interactions between time and each other variable.

Independent variable	“COVID symptoms,” *F* test (*df*)	*P* value	“COVID test,” *F* test (*df*)	*P* value	“COVID vaccine,” *F* test (*df*)	*P* value	“COVID rules,” *F* test (*df*)	*P* value	“COVID lockdown,” *F* test (*df*)	*P* value
Wastewater SARS-CoV-2 prevalence	2.211 (1, 54)	.34	0.418 (1, 54)	.69	28.838 (1, 54)	.002	0.583 (1, 54)	.69	2.834 (1, 54)	.31
Time	0.189 (1, 53)	.88	34.716 (1, 53)	.002	0.120 (1, 53)	.88	4.414 (1, 53)	.12	81.453 (1, 53)	.002
National COVID-19 cases reported	77.157 (1, 52)	.002	28.501 (1, 52)	.002	4.122 (1, 52)	.11	0.677 (1, 52)	.41	7.315 (1, 52)	.03
National COVID-19–related deaths	2.373 (1, 51)	.22	13.42 (1, 51)	.003	18.621 (1, 51)	.003	30.232 (1, 51)	.002	1.193 (1, 51)	.24
Vaccines administered nationally	17.880 (1, 50)	.002	21.308 (1, 50)	.002	8.766 (1, 50)	.02	0.586 (1, 50)	.43	5.770 (1, 50)	.048
Time: wastewater prevalence	0.284 (1, 49)	.98	0.067 (1, 49)	.98	0.011 (1, 49)	.98	0.243 (1, 49)	.98	0.165 (1, 49)	.98
Time: cases	3.349 (1, 48)	.16	15.165 (1, 48)	.002	10.632 (1, 48)	.004	15.869 (1, 48)	.002	1.301 (1, 48)	.27
Time: deaths	3.536 (1, 47)	.18	4.113 (1, 47)	.15	3.04 (1, 47)	.18	0.246 (1, 47)	.59	15.155 (1, 47)	.004
Time: vaccines	0.241 (1, 46)	.81	0.171 (1, 46)	.81	6.898 (1, 46)	.06	1.89 (1, 46)	.37	5.903 (1, 46)	.07

National case data significantly related to Google Trends data for “COVID symptoms” (LM: *t*_54_=7.248, *P*<.001; Figure S4 in Multimedia Appendix 1), “COVID test” (LM: *t*_54_=6.070, *P*<.001; Figure S5 in Multimedia Appendix 1), and “COVID vaccine” (LM: *t*_54_=–3.301, *P*=.002; Figure S6 in Multimedia Appendix 1 but not qPCR-based wastewater SARS-CoV-2 prevalence (LM: *t*_54_=1.360, *P*=.18 Figures S2-6 in Multimedia Appendix 1) nor Google Trends data for “COVID lockdown” (LM: *t*_54_=0.897, *P*=.37; Figure S2 in Multimedia Appendix 1) and “COVID rules” (LM: *t*_54_=0.320, *P*=.75; Figure S3 in Multimedia Appendix 1). Notably, wastewater SARS-CoV-2 RNA prevalence-based predictions significantly positively correlated with the number of reported cases (Spearman ρ=0.274; *P*=.008). Of the search terms included, case data correlated with predictions based on “COVID symptoms” (Spearman ρ=0.683; *P*<.001), “COVID test” (Spearman ρ=0.706; *P*<.001), and “COVID rules” (Spearman ρ=0.409; *P*<.001). National case data significantly related to case numbers predicted by “COVID symptoms” (GLM: *t*_92_=5.158, *P*<.001) and “COVID test” (GLM: *t*_92_=–4.997, *P*<.001) RSVs, but these relationships changed over time (“COVID symptoms”: *t*_92_=–5.162, *P*<.001; “COVID test”: *t*_92_=5.029, *P*<.001; Figure 4). National case data marginally insignificantly related to case numbers predicted by qPCR wastewater SARS-CoV-2 prevalence (GLM: *t*_92_=–1.896, *P*=.02) and “COVID rules” RSVs (GLM: *t*_92_=1.853, *P*=.07), but these relationships were marginally insignificantly related to time (qPCR: *t*_92_=1.920, *P*=.06; “COVID rules”: *t*_92_=–1.866, *P*=.07; Figure 4).

**Figure 3:**
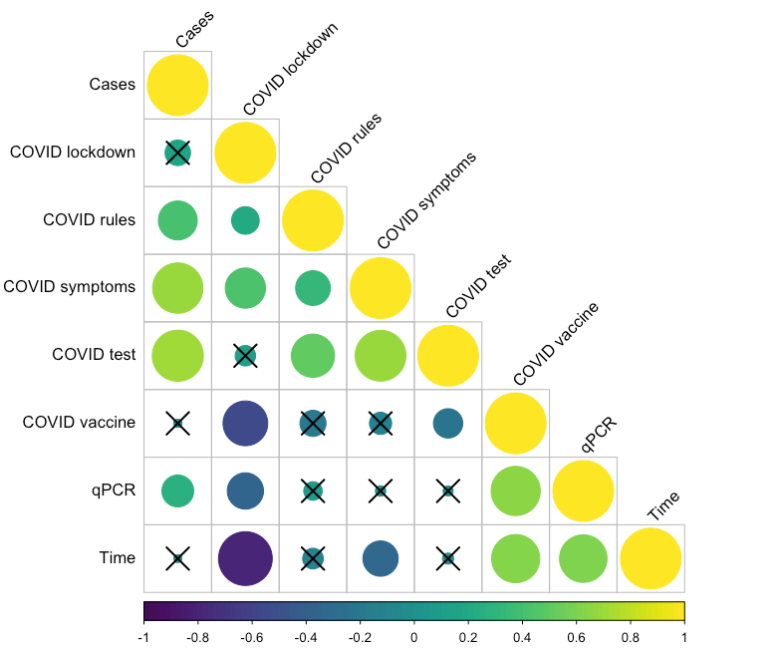
Correlogram of time (study week, ie, progressive number of weeks into the study period), nationally reported cases, and the number of cases predicted based on linear models of cases against Google Trends search volumes and qPCR-based wastewater SARS-CoV-2 prevalence. Circle size and color (purple, through teal to yellow—denoting negative through neutral to positive) indicate the extent and directionality of the correlation. Crossed-out circles are those for which correlations were not significant. qPCR: quantitative polymerase chain reaction.

**Figure 4:**
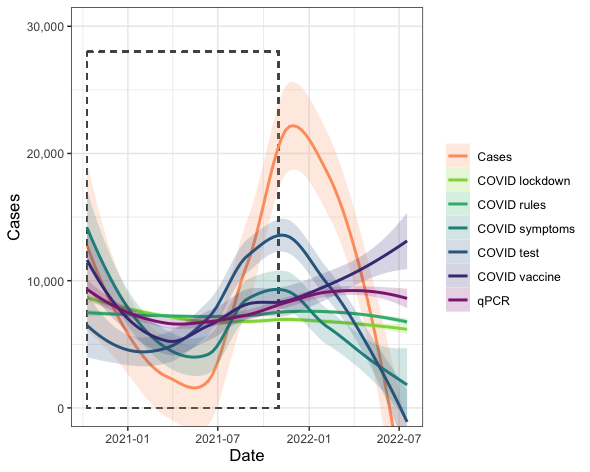
COVID-19 case numbers, and predicted case numbers interpolated and extrapolated based on linear models of case numbers and, separately, each Google Trends search term and qPCR-based SARS-CoV-2 prevalence in wastewater. The dashed rectangle denotes the primary study period, within which data are interpolated. Interpolations are based on data from the primary study period from which models were generated. Extrapolations (outside of the rectangle) are based on data from the following 9 months. Wastewater qPCR-estimated SARS-CoV-2 prevalence is given in light purple, Google Trends data are given in green or blue, and national reported case data are given in orange. Nonsmoothed data are presented in Figure S7 in Multimedia Appendix 1. qPCR: quantitative polymerase chain reaction.

## Discussion

### Principal Findings

This study provides evidence to suggest that public interest in topics related to the pandemic changed dynamically across the study period, with some relation to the prevalence of the virus in wastewater and the number of reported cases. Both internet search volume and qPCR-based SARS-CoV-2 RNA prevalence data provide some predictive potential for monitoring SARS-CoV-2 and could be applied across other contexts.

During the course of this study, comprising 2 significant waves of the COVID-19 pandemic in Wales, the correlation between reported COVID-19 cases and wastewater-quantified SARS-CoV-2 prevalence was significantly positive overall, as has been demonstrated in previous studies [28,34], but this correlation may have changed over time. Comparing the prevalence of wastewater SARS-CoV-2 estimates and national cases across the full study period shows that wastewater prevalence of SARS-CoV-2 peaked substantially higher in October 2020 than the rest of the study period, whereas case data peaked the following October (Figure S1 in Multimedia Appendix 1). Indications of correlation between SARS-CoV-2 prevalence in wastewater and COVID-19 disease prevalence were recognized at an early stage of the pandemic in other countries [32]. The Google Trends search volume data show web-based searching for some COVID-19–related strings largely reduced over time, although this was highly dependent on the search string. This could indicate reduced public interest, fluctuations that were reported even in the initial months of the pandemic despite the importance of sustained public action to ensure the success of public health measures [50].

In this same period, many of the search volumes, with the intuitive exception of “COVID vaccine,” appear to inversely correlate with increased vaccinations. This suggests that the public may have been seeking vaccine opportunities and otherwise expressed less interest in COVID-19 following mass vaccinations, although additional data would be required to confirm this. Importantly, searches for “COVID vaccine” may also represent those that were concerned with misinformation or conspiracy theories that were commonplace, particularly around the vaccine [11].

The search term “COVID test” was maintained at a relatively constant level throughout the study and, along with “COVID symptoms” and “COVID vaccine,” correlated with the wastewater SARS-CoV-2 prevalence just as national case data did. This indicates the potential of carefully selected search terms for estimating the prevalence of the virus, further ratified by the predictions made in this study. The relationship between predictions and case data varied greatly depending on the data used to guide predictions and, importantly, these relationships changed over time. The variable potential of infoveillance to predict epidemiological trends has been recorded in other cases, such as for Google Flu Trends [13,15], and is an important consideration for the use of infoveillance in a monitoring context. The efficacy of infoveillance is contingent on public interest consistently reflecting epidemiology, which is ultimately unlikely for global pandemics given natural spikes and fluctuations in public interest. It is, however, important to contextualize this with the likely reasons for members of the public searching with this particular string. Search volume data could nonetheless provide anecdotal monitoring of disease prevalence, especially since many nations face difficulties in monitoring the virus using molecular methods or population-level testing. Search volume data, while imperfect, may provide a valuable alternative for anecdotal epidemiological monitoring in nations or regions lacking access to alternatives [51], but the search terms must be carefully considered, closely monitored, and interpreted with appropriate skepticism.

The strong positive correlation between national testing, wastewater monitoring data, and Google RSVs has previously been demonstrated in the United States [34]. The relation of search term data to SARS-CoV-2 prevalence in wastewater changed over time, suggesting that such approaches require monitoring and constant evaluation, again suggesting that an approach combining data types may be optimal [34]. Importantly, the predictions made based on qPCR-based wastewater monitoring were marginally insignificantly related to recorded cases. Given the relative objectivity of this molecular monitoring, this is likely to reflect the inconsistent accuracy of national case data reporting as the pandemic progressed, highlighting the need for objective measures of virus prevalence irrespective of public participation. While these different data types dynamically interact and often imperfectly reflect one another, as demonstrated by our univariate predictions, together they could generate models with greater predictive power for forecasting improved above that of univariate approaches [34]. This aligns with the “One Health” perspective of integrating different data types across disciplinary boundaries to monitor health care and epidemiological events more holistically [22,23]. Wastewater monitoring has been integrated into One Health frameworks for pathogen monitoring [52] and emerging concepts such as antimicrobial resistance in the environment [53]. Given that infoveillance similarly aligns with the principles of One Health [23], this presents an ideal opportunity to integrate different data types for sociobiological monitoring of SARS-CoV-2 and other pandemic agents.

### Limitations

Regarding infoveillance, this study relied exclusively on Google search volume data; while this represents the most used search engine and thus the greatest single source of such data, other search engines are regularly used that might provide different insights. Web-based search data, while an asset for assessing public responses, is also collected without the context of its users’ motives; thus, assumptions cannot reliably be made about the specific interests related to each search string. Even without this context, however, the search volumes presented in this study indicate interest, positive or negative, in those topics. Previous studies have demonstrated that the efficacy of these data in predicting epidemiological trends can be, at best, variable and, at worst, ineffective [13-15]; this can be mitigated to some degree via robust statistical methods to increase the reliability and accuracy of infoveillance for epidemiological “nowcasting” [15], but the integration of these data into more holistic frameworks across disciplinary boundaries could further ameliorate these inaccuracies and provide increasingly accurate predictions [22,23].

While the qPCR data in this study represent a nationwide effort to monitor SARS-CoV-2, they do not comprehensively cover the nation of Wales, which is otherwise fully represented by the Google Trends and national reporting data. Importantly, the qPCR data do account for all of South Wales, which, in turn, accounts for approximately 71% of the national population [54], meaning that these data should accurately reflect the overall national SARS-CoV-2 prevalence. Future studies could investigate how different spatiotemporal resolutions of data affect the accuracy and outcomes of analyses such as these, especially given that this will impact the feasibility of long-term monitoring using most methods.

The progression of COVID-19 as a global pandemic continues to be extremely complicated and unpredictable, and the findings of this study focus on just 1 period in this evolving situation, prior to the emergence of the SARS-CoV-2 Omicron variant and its sublineages. More importantly, the early months of the pandemic are not represented due to the unavailability of qPCR data for that period. While this study relates primarily to those later months of the first year of the pandemic through to the second year, the use of Google Trends data may have been more powerful in the early months of the pandemic when public familiarity was lower and more people were seeking information.

### Conclusions

Both molecular monitoring of wastewater and infoveillance approaches demonstrate potential for monitoring and prediction of epidemiological trends. Personal testing and surveys can introduce latency to monitoring, lack randomization, and can receive reduced participation for fear of positive test outcomes [10]; thus, reduced dependency on these data through widespread adoption of wastewater monitoring will likely improve the accuracy of epidemiological data. Wastewater monitoring has previously correlated strongly with national case data [32], but any decrease in this correlation must importantly be viewed with respect to the public interest and how this might impact reported case data. Disease surveillance via wastewater monitoring provides many potential benefits, not least its objectivity. As public interest in the pandemic wanes, widespread molecular analysis of wastewater will become increasingly important as personal testing data become increasingly inaccurate at the population level. Public access to wastewater monitoring data has been facilitated through web-based reporting, including the data used in this study [38], but accessible presentation of these data in interactive dashboards, as has been the case for other national data, may increase public understanding, appreciation, and use of this important data source.

## Appendix

Multimedia Appendix 1 Supplementary materials.

## Abbreviations

CDC: Centers for Disease Control and Prevention
GLM: generalized linear model
LM: linear model
MLM: multivariate linear model
qPCR: quantitative polymerase chain reaction
RSV: relative search volume
RT: reverse transcription
RT-qPCR: quantitative reverse transcription polymerase chain reaction
