# Supplementary material for: Monitoring SARS-CoV-2 Using Infoveillance, National Reporting Data, and Wastewater in Wales, United Kingdom: Mixed Methods Study
Source: JMIR Infodemiology. 2023 Nov 23;3:e43891. doi: 10.2196/43891 (PMC10669927; doi:10.2196/43891)

**Table S1.** Correlation analysis results for time (study week, ie, progressive number of weeks into the study period), Google Trends search volumes, nationally reported cases, deaths and vaccines, and wastewater SARS-CoV-2 RNA prevalence.

| Variable 1 | Variable 2 | Spearman ρ | *P* value |
| --- | --- | --- | --- |
| COVID rules | COVID symptoms | 0.147 | .280 |
| COVID rules | COVID lockdown | 0.181 | .182 |
| COVID symptoms | COVID lockdown | 0.194 | .152 |
| COVID rules | COVID test | 0.125 | .361 |
| COVID symptoms^a^ | COVID test^a^ | 0.581 | <.001 |
| COVID lockdown^a^ | COVID test^a^ | –0.539 | <.001 |
| COVID rules | COVID vaccine | –0.006 | .967 |
| COVID symptoms^a^ | COVID vaccine^a^ | –0.517 | <.001 |
| COVID lockdown | COVID vaccine | 0.141 | .302 |
| COVID test^a^ | COVID vaccine^a^ | –0.39 | .003 |
| COVID rules | Cases | –0.099 | .467 |
| COVID symptoms^a^ | Cases^a^ | 0.805 | <.001 |
| COVID lockdown | Cases | 0.073 | .593 |
| COVID test^a^ | Cases^a^ | 0.531 | <.001 |
| COVID vaccine^a^ | Cases^a^ | –0.495 | <.001 |
| COVID rules | Deaths | –0.124 | .363 |
| COVID symptoms^a^ | Deaths^a^ | 0.5 | <.001 |
| COVID lockdown^a^ | Deaths^a^ | 0.61 | <.001 |
| COVID test | Deaths | –0.179 | .187 |
| COVID vaccine | Deaths | –0.081 | .553 |
| Cases^a^ | Deaths^a^ | 0.646 | <.001 |
| COVID rules | Vaccines | –0.154 | .259 |
| COVID symptoms^a^ | Vaccines^a^ | –0.795 | <.001 |
| COVID lockdown^a^ | Vaccines^a^ | –0.273 | .042 |
| COVID test^a^ | Vaccines^a^ | –0.298 | .026 |
| COVID vaccine^a^ | Vaccines^a^ | 0.516 | <.001 |
| Cases^a^ | Vaccines^a^ | –0.675 | <.001 |
| Deaths^a^ | Vaccines^a^ | –0.552 | <.001 |
| COVID rules | qPCR | –0.109 | .425 |
| COVID symptoms^a^ | qPCR^a^ | 0.369 | .005 |
| COVID lockdown | qPCR | –0.05 | .717 |
| COVID test^a^ | qPCR^a^ | 0.356 | .007 |
| COVID vaccine^a^ | qPCR^a^ | –0.504 | <.001 |
| Cases^a^ | qPCR^a^ | 0.428 | .001 |
| Deaths | qPCR | 0.044 | .746 |
| Vaccines^a^ | qPCR^a^ | –0.299 | .025 |
| COVID rules | Time | –0.249 | .065 |
| COVID symptoms | Time | –0.112 | .411 |
| COVID lockdown^a^ | Time^a^ | –0.829 | <.001 |
| COVID test^a^ | Time^a^ | 0.638 | <.001 |
| COVID vaccine | Time | –0.096 | .481 |
| Cases | Time | 0.132 | .332 |
| Deaths^a^ | Time^a^ | –0.492 | <.001 |
| Vaccines^a^ | Time^a^ | 0.337^a^ | .011^a^ |
| qPCR | Time | 0.157 | .249 |

^a^Significant correlations.

**Table S2.** Correlation analysis results for nationally reported case data, time (study week, ie, progressive number of weeks into the study period), and predicted cases based on Google Trends search volumes and wastewater SARS-CoV-2 RNA prevalence.

| Variable 1 | Variable 2 | Spearman ρ | *P* value |
| --- | --- | --- | --- |
| Cases^a^ | qPCR^a^ | 0.274 | .008 |
| Cases^a^ | COVID symptoms^a^ | 0.683 | <.001 |
| Cases | COVID lockdown | 0.176 | .091 |
| Cases^a^ | COVID rules^a^ | 0.409 | <.001 |
| Cases | COVID vaccine | –0.019 | .860 |
| Cases^a^ | COVID test^a^ | 0.706 | <.001 |
| Cases | Time | –0.017 | .871 |
| COVID lockdown^a^ | COVID rules^a^ | 0.206 | .047 |
| COVID lockdown^a^ | COVID vaccine^a^ | –0.541 | <.001 |
| COVID lockdown | COVID test | 0.113 | .280 |
| COVID lockdown^a^ | Time^a^ | –0.782 | <.001 |
| COVID rules | COVID vaccine | –0.185 | .077 |
| COVID rules^a^ | COVID test^a^ | 0.505 | <.001 |
| COVID rules | Time | –0.116 | .270 |
| COVID symptoms^a^ | COVID lockdown^a^ | 0.437 | <.001 |
| COVID symptoms^a^ | COVID rules^a^ | 0.326 | .001 |
| COVID symptoms | COVID vaccine | –0.133 | .204 |
| COVID symptoms^a^ | COVID test^a^ | 0.697 | <.001 |
| COVID symptoms^a^ | Time^a^ | –0.337 | .001 |
| COVID test | Time | –0.034 | .749 |
| COVID vaccine^a^ | COVID test^a^ | –0.233 | .025 |
| COVID vaccine^a^ | Time^a^ | 0.626 | <.001 |
| qPCR | COVID symptoms | 0.029 | .779 |
| qPCR^a^ | COVID lockdown^a^ | –0.356 | <.001 |
| qPCR | COVID rules | 0.093 | .376 |
| qPCR^a^ | COVID vaccine^a^ | 0.654 | <.001 |
| qPCR | COVID test | 0.028 | .793 |
| qPCR^a^ | Time^a^ | 0.611 | <.001 |

^a^Significant correlations.

**Figure S1.** Relative search volumes extracted from Google Trends compared against nationally reported data and qPCR-based estimates of prevalence for SARS-CoV-2. All values are normalized so that the maximum value for each variable is 100. Dashed rectangles represent periods of national lockdown for reference. Wastewater qPCR-estimated SARS-CoV-2 prevalence is given in light purple, Google Trends data are given in green/blue, and national data are given in orange/red/purple. Smoothed data are presented in Figure 2.


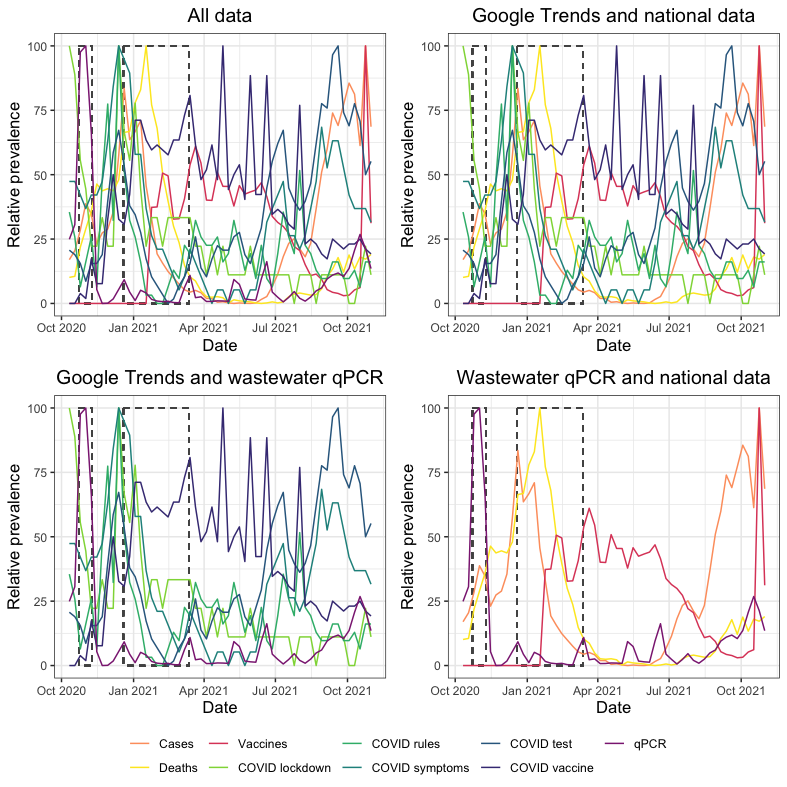


**Figure S2.** Paired plots showing the relationships between nationally reported case data, qPCR-based wastewater SARS-CoV-2 prevalence, and Google Trends RSVs for “COVID lockdown”.


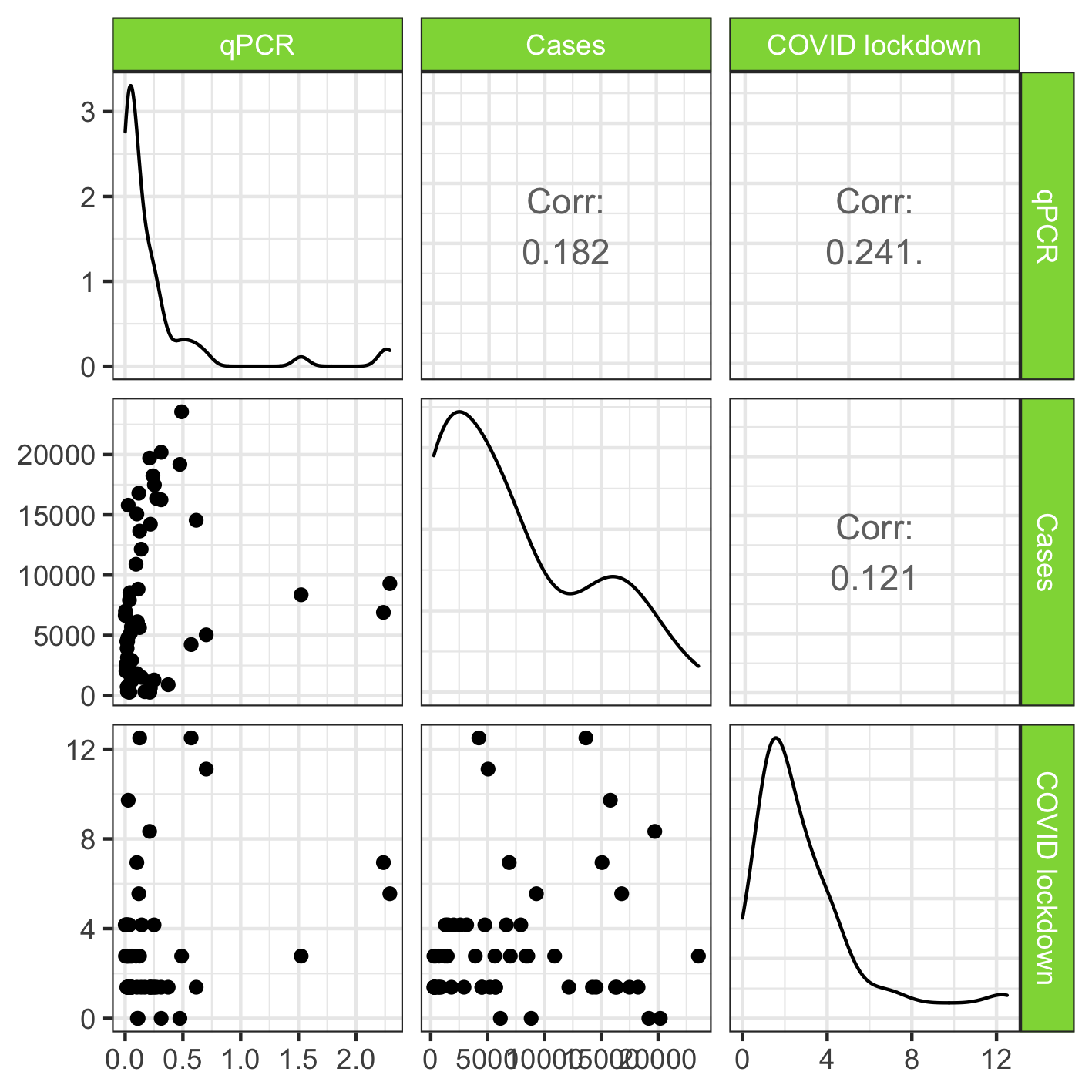


**Figure S3.** Paired plots showing the relationships between nationally reported case data, qPCR-based wastewater SARS-CoV-2 prevalence, and Google Trends RSVs for “COVID rules”.


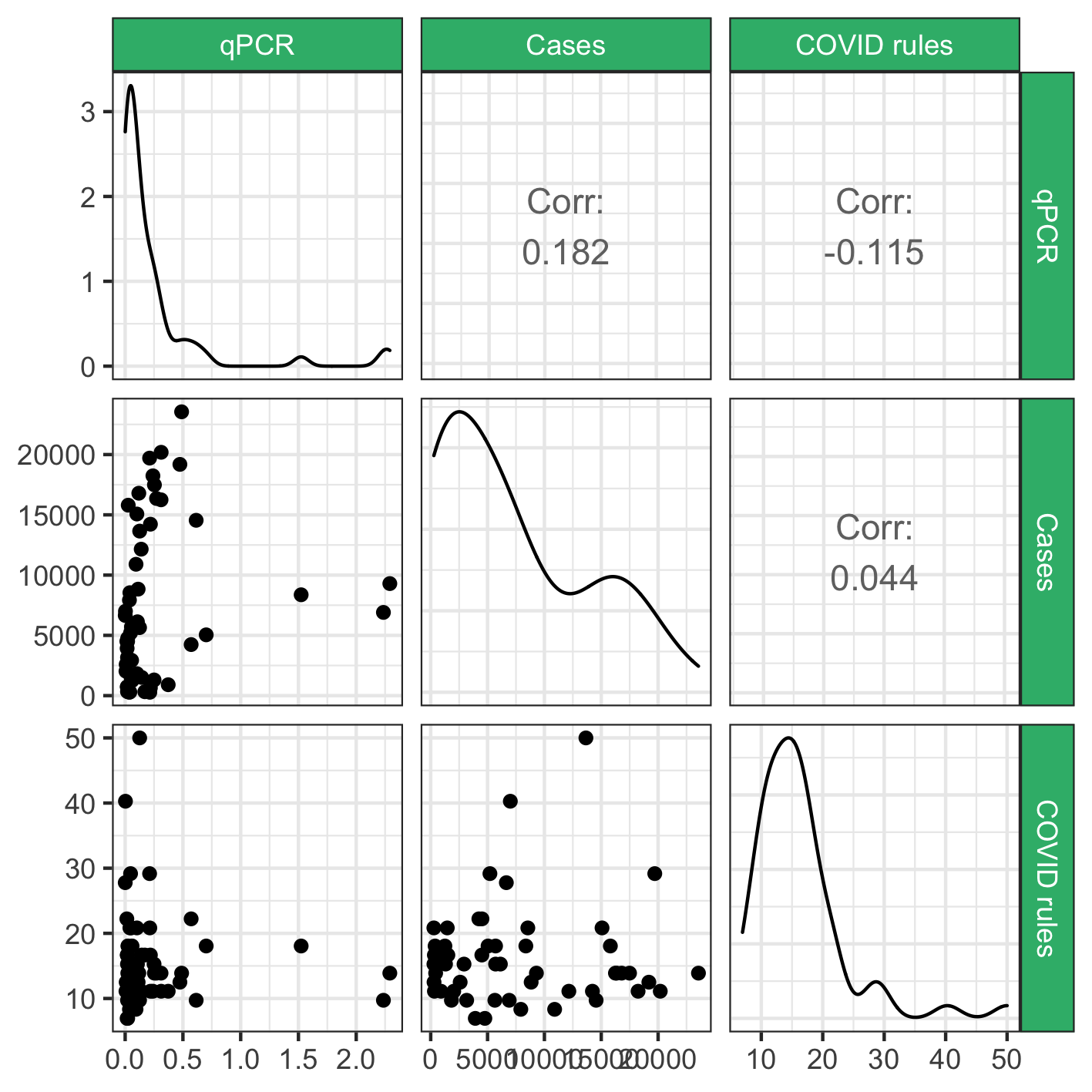


**Figure S4.** Paired plots showing the relationships between nationally reported case data, qPCR-based wastewater SARS-CoV-2 prevalence, and Google Trends RSVs for “COVID symptoms”.


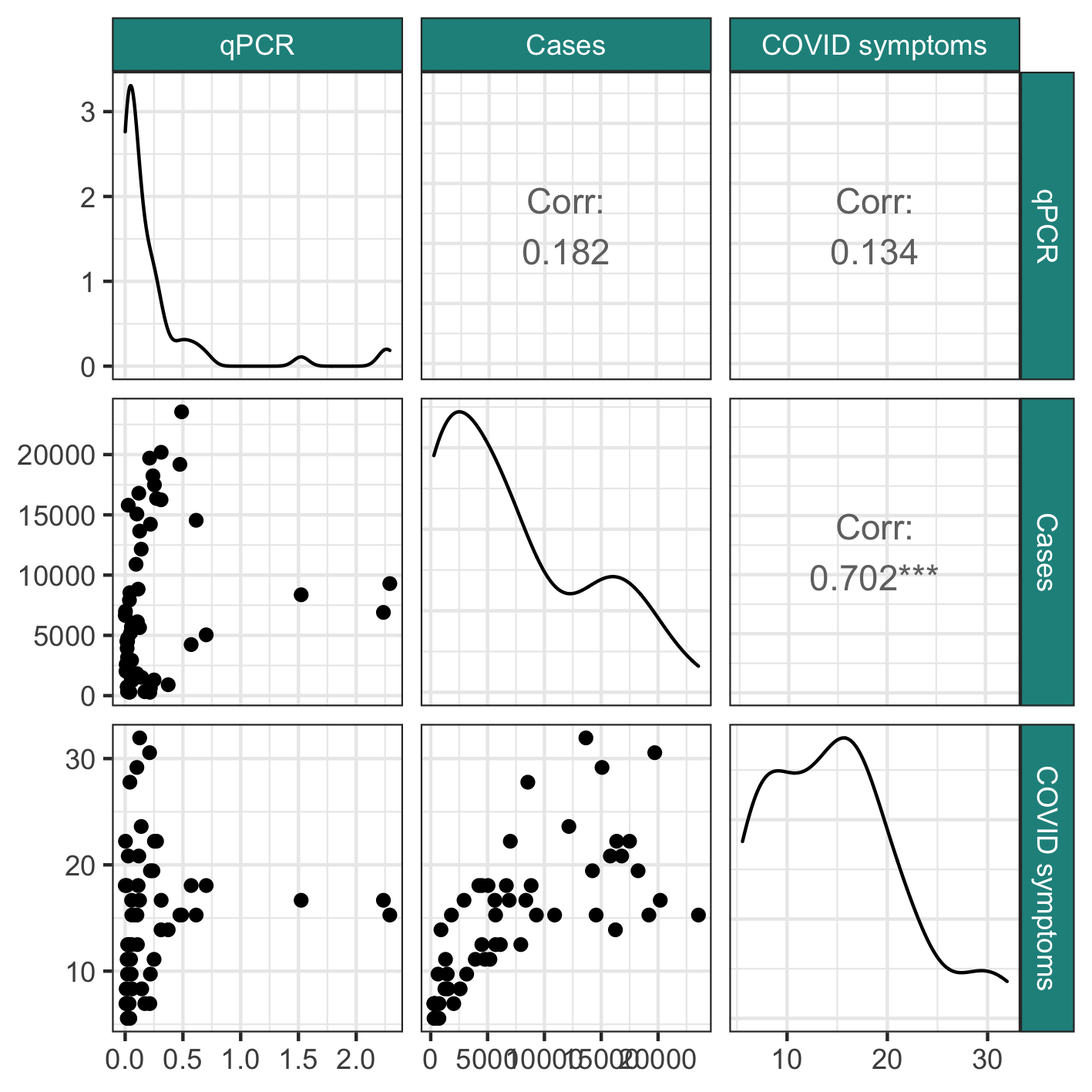


**Figure S5.** Paired plots showing the relationships between nationally reported case data, qPCR-based wastewater SARS-CoV-2 prevalence, and Google Trends RSVs for “COVID test”.


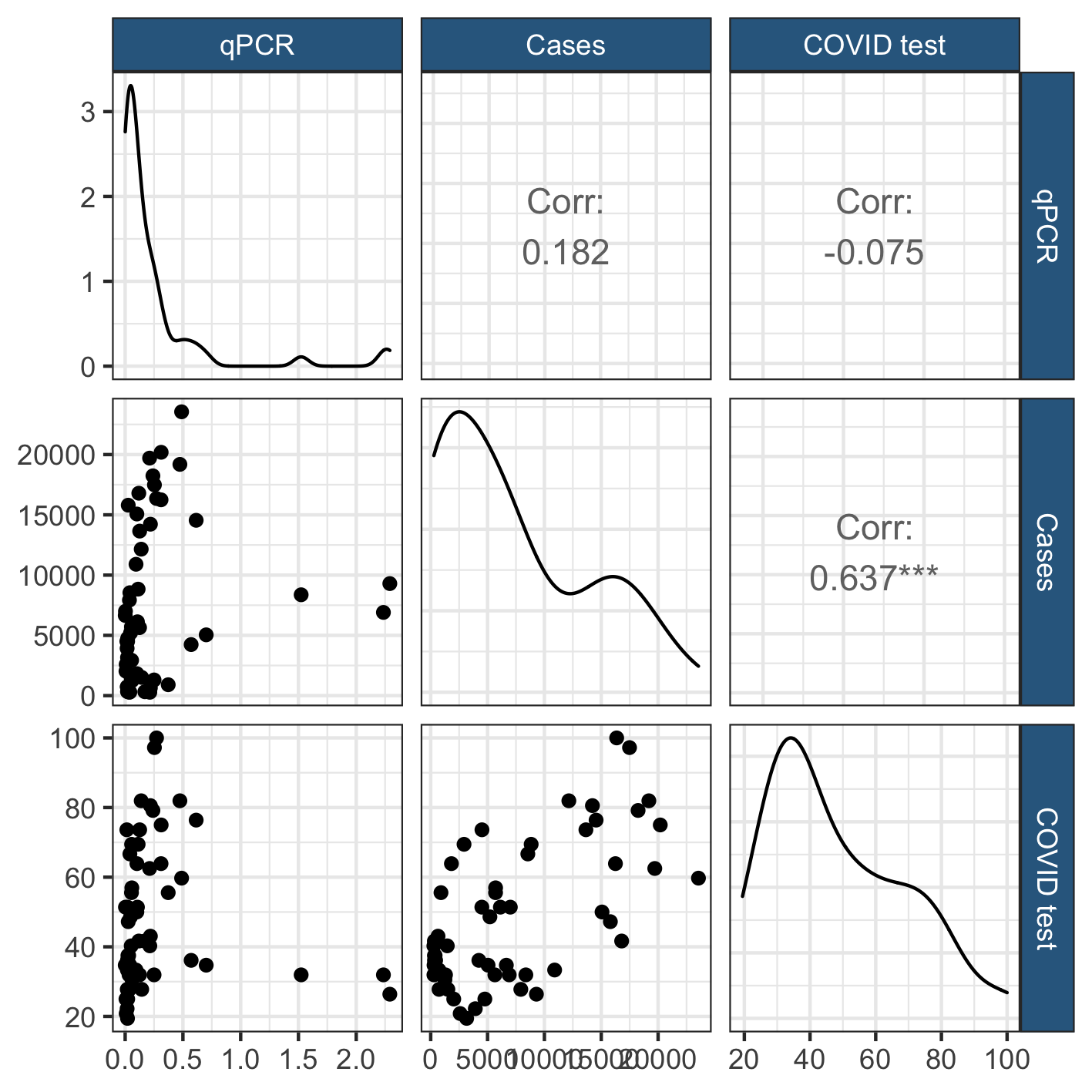


**Figure S6.** Paired plots showing the relationships between nationally reported case data, qPCR-based wastewater SARS-CoV-2 prevalence, and Google Trends RSVs for “COVID vaccine”.


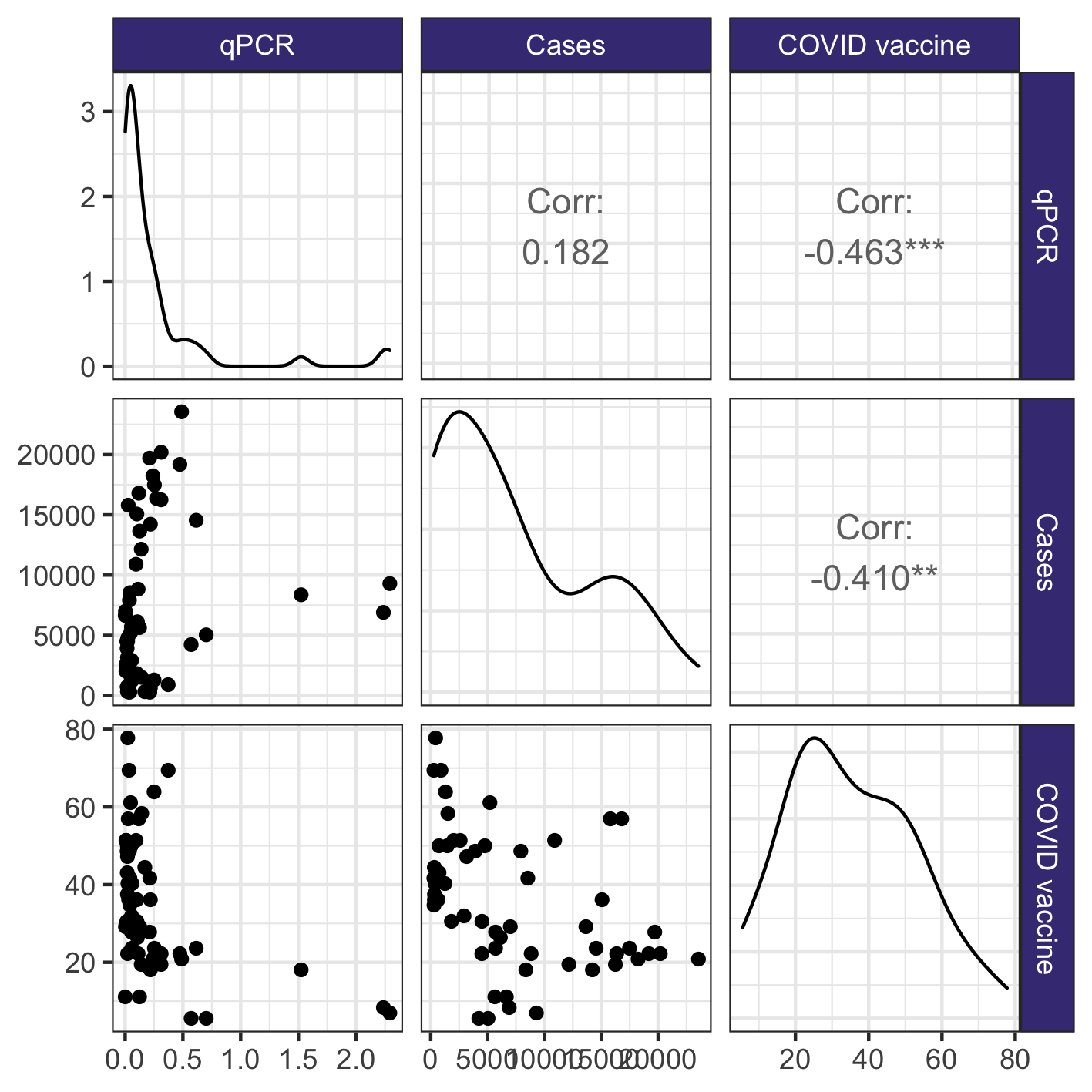


**Figure S7.** COVID-19 case numbers, and predicted case numbers interpolated (solid lines) and extrapolated (dashed lines) based on linear models of case numbers and, separately, each Google Trends search term and qPCR-based estimates of SARS-CoV-2 prevalence in wastewater. Interpolations are based on data from the primary study period from which models were generated. Extrapolations are based on data from the following 9 months. The dashed rectangle also denotes the primary study period. Wastewater qPCR-estimated SARS-CoV-2 prevalence is given in light purple, Google Trends data are given in green/blue, and national reported case data are given in orange. Smoothed data are presented in Figure 4.


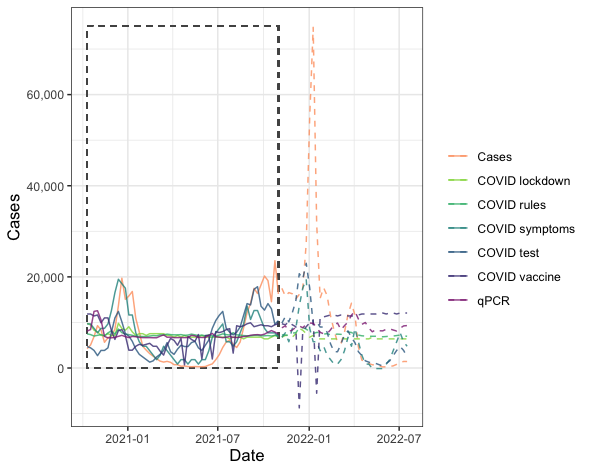

Supplement: Multimedia Appendix 1 [file infodemiology_v3i1e43891_app1.docx]
